# Repurpose terbutaline sulfate for amyotrophic lateral sclerosis using electronic medical records

**DOI:** 10.1038/srep08580

**Published:** 2015-03-05

**Authors:** Hyojung Paik, Ah-Young Chung, Hae-Chul Park, Rae Woong Park, Kyoungho Suk, Jihyun Kim, Hyosil Kim, KiYoung Lee, Atul J. Butte

**Affiliations:** 1 Department of Biomedical Informatics, Ajou University School of Medicine, Suwon, Gyeonggido, Korea; 2 Graduate School of Medicine, Korea University, Ansan, Gyeonggido, Korea; 3 Department of Pharmacology, Brain Science & Engineering Institute, Kyungpook National University, Daegu, Korea; 4 Division of Systems Medicine, Department of Pediatrics, Stanford University School of Medicine, Stanford, and Lucile Packard Children's Hospital, Palo Alto, CA, USA

## Abstract

Prediction of new disease indications for approved drugs by computational methods has been based largely on the genomics signatures of drugs and diseases. We propose a method for drug repositioning that uses the clinical signatures extracted from over 13 years of electronic medical records from a tertiary hospital, including >9.4 M laboratory tests from >530,000 patients, in addition to diverse genomics signatures. Cross-validation using over 17,000 known drug–disease associations shows this approach outperforms various predictive models based on genomics signatures and a well-known “guilt-by-association” method. Interestingly, the prediction suggests that terbutaline sulfate, which is widely used for asthma, is a promising candidate for amyotrophic lateral sclerosis for which there are few therapeutic options. *In vivo* tests using zebrafish models found that terbutaline sulfate prevents defects in axons and neuromuscular junction degeneration in a dose-dependent manner. A therapeutic potential of terbutaline sulfate was also observed when axonal and neuromuscular junction degeneration have already occurred in zebrafish model. Cotreatment with a β_2_-adrenergic receptor antagonist, butoxamine, suggests that the effect of terbutaline is mediated by activation of β_2_-adrenergic receptors.

Discovery of unknown indications or biological targets for approved drugs (i.e., drug repositioning) has several advantages over new drug development, especially when reusing drugs with known safety profiles. The precise prediction of new therapeutic indications using computational methods could accelerate the drug-development process and has been used to generate new repositioning opportunities^1^. Including our own previous attempt^2^, gene expression-based computational discoveries typically originate from an analysis of the molecular signatures of drugs and diseases^3^. Integrating various types of genomics information has also been used for computational analysis of drug repositioning^4^. Previous studies to reposition drugs have exploited the relationships between drugs and diseases based on related molecular information.

Recently, a few studies have applied phenotypic profiling of the entire human system, such as drug-induced side effects, for drug repositioning^5^. Long-term observations of the therapeutic effects of arsenic trioxide yielded a new indication for acute promyelocytic leukemia^6^. Meanwhile, we and others have proposed a method based on a “guilt-by-association” (GBA) approach, which uses the known therapeutic indications of drugs to predict new indications based on pairwise relationships between diseases and associated sets of drugs^7^. However, to our knowledge, extensive and direct clinical cohort-based approaches to drug repositioning, such as the physiological and phenotypic screening of diseases and drugs in human individuals, have not yet been attempted.

Electronic medical records (EMRs) contain digitally recorded medical and pathophysiological data, including the results of laboratory tests of serum, urine, and other samples, e.g., the blood glucose levels in diabetic patients. As indicated in our previous study, analysis of laboratory test results in EMRs can determine the clinical character of diseases and their responses to drugs^8^,^9^,^10^. Here, we describe the development of a generalized method for drug repositioning, which uses the laboratory test results from EMRs, in addition to genomics signatures from public resources. As a proof-of-concept, we applied this approach to reposition a drug widely approved for asthma, to amyotrophic lateral sclerosis (ALS) and validated the approach with experiments in a model of ALS.

## Results

### Drug repositioning using electronic medical and genomics data

**Figure 1:**
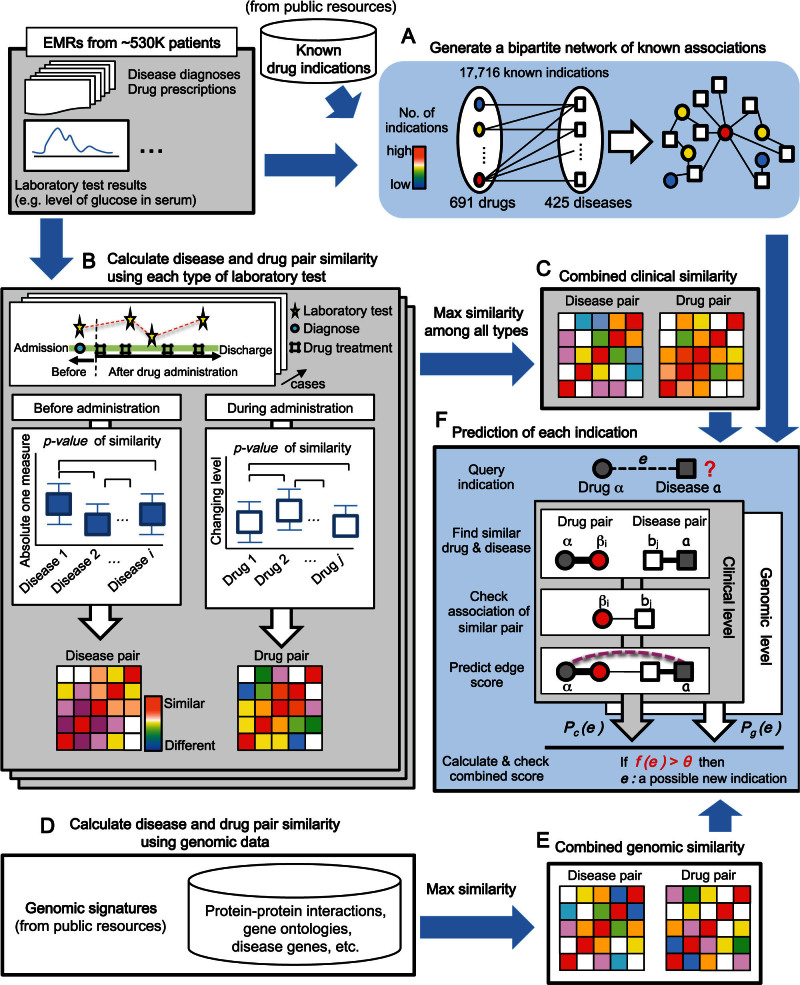
Overview of ClinDR. (A) Construction of a drug–disease network. Known associations between drugs (circle nodes) and target diseases (square nodes) are represented as a bipartite network (black lines). We utilized existing drug prescription records in our EMRs and public drug indication resources to generated standard known drug-disease associations. (B) Calculation of drug–drug and disease–disease similarities using clinical signatures, such as distribution or pattern of laboratory test results under drugs or diseases related conditions. For disease pair similarity ClinDR uses the absolute values of individual types of laboratory test performed before any drug treatment. For drug pair similarity, ClinDR uses the changing pattern of laboratory test results during the corresponding drug medication. Then, ClinDR finds the maximum similarity scores across diverse types of laboratory test (C). (D–E) Calculation of drug–drug and disease–disease similarities using genomic signatures. (F) Prediction of final score (*f(e)* > *θ*, *true*) between the query indication (i.e. between drug α and disease a) using the combined clinic and genomic similarity matrixes from (C) and (E). The similarities between drug pairs or disease pairs are represented as edge widths. *P_c_(e)* and *P_g_(e)*: the maximum score of a query indication (*e*) using clinical (*P_c_(e)*) and genomic (*P_c_(e)*) data, respectively. β_i_: a similar drug to α. b_i_: a similar disease to a.

We designed a novel algorithm for drug repositioning, referred to as clinical and genomics signature-based prediction for drug repositioning (ClinDR) that utilizes both clinical data from EMRs and genomics data from public resources (Table 1). We used the clinical profiles of drug-treated and diseased patients in a 13-year EMR dataset from the tertiary teaching hospital of Ajou University. The objective of ClinDR is to identify hitherto unknown indications for drugs used to treat known diseases using known drug–disease associations for similar drugs and diseases. In this model, the basic assumption is that similar drugs can be used to treat similar diseases. We first represented known drug–disease associations as a bipartite network where diseases or drugs are nodes, and the edges between them represent potential therapeutic drug use (Fig. 1A). For known drug–disease associations, we combined the drug medication records in our EMR database and known indications from a public database^11^. In summary, 691 drug nodes, 425 disease nodes, and 17,716 edges for drug–disease associations were prepared.

To calculate the disease–disease similarities at a clinical level, we compared the distributions of laboratory test results between disease pairs before any drug administration (Methods; Fig. 1B). Using each type of laboratory test, we computed a *p*-value for the distribution of results between disease pairs using a Wilcoxon rank-sum test. In this test, stronger *p*-values for results between disease pairs indicate higher similarity for laboratory test results between the diseases. For the drug–drug similarities, we used the degree of change in laboratory test results after administration of individual drugs. Subsequently, we prepared a single similarity matrix for drug–drug or disease–disease pairs by selecting the maximum values for the generated similarities among the diverse types of laboratory tests (Fig. 1C). It is important to note that we normalized the *p*-value similarities using a rank method to reduce heterogeneity across different types of laboratory tests before generation of a single similarity matrix. The main types of laboratory tests based on their coverage of drugs and disease are shown in Table 2.

We also graded the similarities of disease or drug pairs on diverse genomics data including Gene Ontology terms and disease- or drug-related protein networks (Fig. 1D). Genomics level similarities were represented by selecting the highest-ranking normalized *p*-value similarities, such as those for protein interactions (Methods; Fig. 1E).

Using the known drug–disease network and the drug and disease pair similarities at the clinical and genomics levels, ClinDR was used to calculate a final score for each edge between a drug and a disease to determine whether the corresponding edge is a candidate for repositioning (Fig. 1F).

### Analysis of clinical similarities for drug or disease pairs

**Figure 2:**
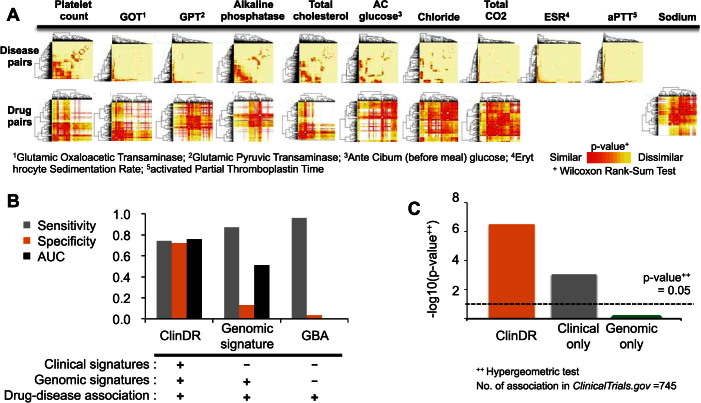
Clustering of drug- or disease-pair similarities of clinical data and performance evaluations. (A) Hierarchical clustering of Wilcoxon rank sum test for disease-disease and drug-drug pairs by distinct laboratory test results. (B) Bar chart for the 10-fold cross-validation of ClinDR with/without clinical physiome signatures and the GBA method. The GBA method presents deterministic results, without AUC. (C) The enrichment test of novel ClinDR repositionings with clinical trials in *ClinicalTrials.gov*.

For drug or disease similarities, we used diverse types of laboratory tests separately to reflect different clinical characteristics of drugs and diseases. Clustering results from the similarities between drugs or between diseases showed that distinct types of laboratory test results produced different groups of related diseases or drugs (Fig. 2A). For example, erythrocyte sedimentation rate (ESR) levels, which are an indicator of inflammation^12^ showed that diseases similar to acute nephritic syndrome (i.e. renal inflammation) included blood cell disorders and infectious diseases, such as leukemia, anemia and mycobacterial infections (Fig. S1A). When ESR levels were used, diseases related to immune mechanisms were clustered together (20 immune related diseases among 22 clustered diseases; hypergeometric test *p* = 4.2 × e^−26^; Fig. S1B). For total cholesterol level, which is widely used to detect metabolic or cardiac abnormalities, diseases related to endocrine and circulatory diseases were clustered together (51 endocrine disorders among 100 clustered diseases; *p* = 3.43 × e^−39^; Fig. S1C). Likewise, changing levels of glutamic oxaloacetic transaminase activity during drug therapy clustered similar drug classes together, including blood-forming organ and cardiovascular system-related drugs (31 drugs are **B** or **C** drug class of Anatomical Therapeutic Chemical classification system (ATC) codes among 45 clustered drugs; *p* = 3.17 × e^−12^; Fig. S1D).

### ClinDR performance assessment

We evaluated the performance of ClinDR against other methods. We used (i) the complete set of ClinDR features; (ii) ClinDR using only genomics signatures; and (iii) a GBA algorithm^7^ based on a tenfold cross-validation scheme using 17,716 known associations. ClinDR outperformed other methods, more so when it included the clinical signatures of drugs and diseases (Fig. 2B). Using a threshold (final score > 0.9), we found 3,891 new indications for 226 drugs and 55 diseases that were previously not known to be associated (Fig. S2). The new indications had a high degree of overlap with current clinical trials for discovery of new indications in *ClinicalTrials.gov* (*p* = 3.0 × e^−07^; Fig. S3) and the overlap was higher than that found for other predictive models including ClinDR based on genomics signatures alone (Fig. 2C). Moreover, the predictions covered various classes of drugs (Fig. S4).

**Figure 3:**
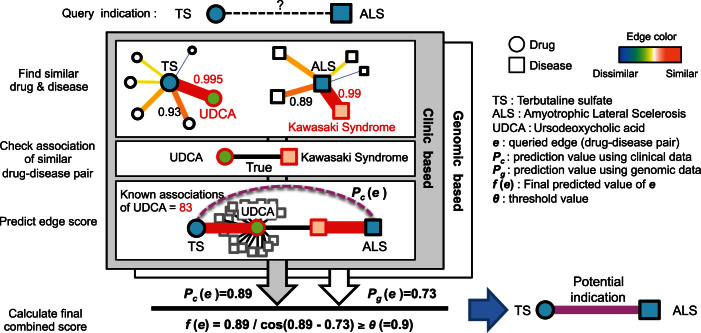
Schematic view for the repurpose prediction of terbutaline sulfate for ALS. ClinDR predict terbutaline sulfate (TS) as a promising candidate for ALS by drug-drug and disease-disease similarity analysis. Presented scores in between TS and Ursodeoxycholic acid (UDCA), and ALS and Kawasaki syndrome were analyzed similarity values using clinical signatures from EMRs (0.995 for the similarity between TS-UDCA pair and 0.99 for the disease pair similarity between ALS and Kawasaki syndrome). By integration of clinical (*P_c_*) and genomic signature based predictions (*P_g_*), TS was determined as a repositioning candidate for ALS therapy.

Among the new indications predicted, one example was terbutaline sulfate (TS) as a potential drug for amyotrophic lateral sclerosis (ALS) treatment. From the EMR-based similarity matrixes, TS displayed the highest similarity with ursodeoxycholic acid (UDCA; similarity = 0.995; Fig. 3). Moreover, Kawasaki syndrome was the most similar disease to ALS (similarity = 0.99). As seen in our EMRs, UDCA has been used to treat Kawasaki syndrome because UDCA regulates apoptosis^13^,^14^. Based on the combined score of clinical and genomics data, ClinDR predicted that TS was the highest ranked candidate for repositioning among all drugs without a former association with ALS (final score > 0.9).

### Terbutaline sulfate as a candidate for ALS therapy

**Figure 4:**
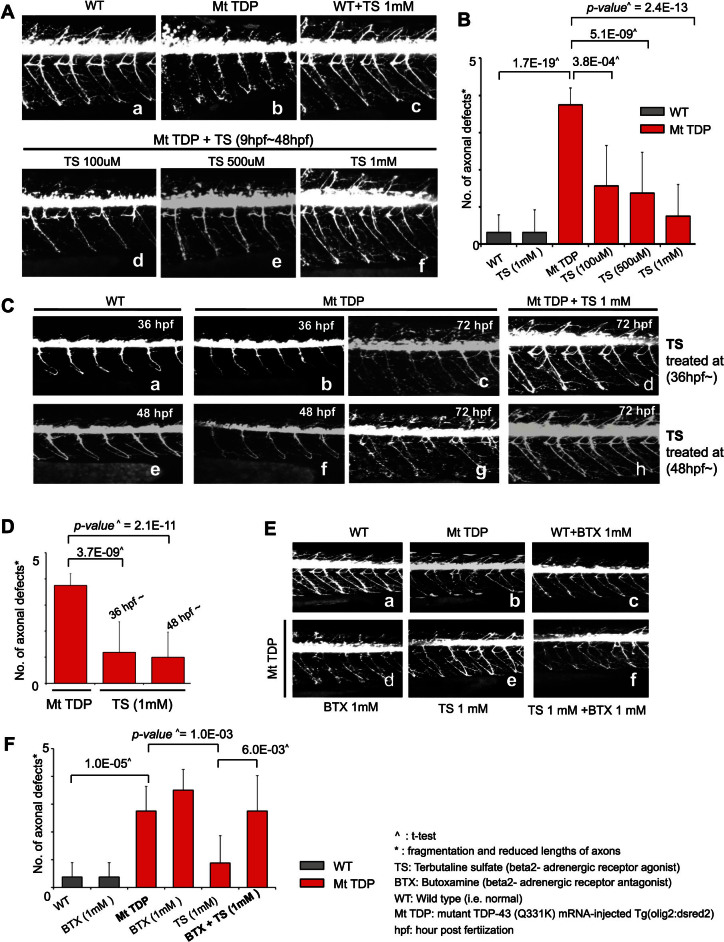
Experimental validation of terbutaline sulfate repurposing for ALS. (A, C, F) All panels show lateral views of *Tg*(*olig2:dsred2*) spinal cords of zebrafishes, with anterior to the left and dorsal to the top. (A) Terbutaline sulfate (TS) prevent motor axon and neuromuscular junction degeneration of ALS model (d–f). In normal conditions, treatment with TS (c) had nonlethal effects compared with the untreated condition (a). Mt TDP indicates mutant TDP-43 mRNA-injected model and WT means wild type (i.e. normal). (B) Statistical analysis of **panel A**. Axonal defects indicate fragmentation and reduced lengths of axons. Data were obtained from 4 myotome segments from each of 10 control and 10 TS-treated models. (C) TS rescues the ALS phenotype. Mt TDPs had abnormal motor axon phenotypes at 36 h postfertilization (hpf) (b) and 48 hpf (f) compared with WTs (a, c). These models had clear motor axon and neuromuscular junction (NMJ) defects at 72 hpf (e, g). Mt TDP with 1 mM TS at 36 hpf (c) and 48 hpf (g), respectively, rescued motor axon and NMJ defects at 72 hpf (d, h). (D) Statistical analysis of **panel C**. (E) Inhibition of therapeutic effect of TS by beta2-adrenergic receptor antagonist, Butoxamine (BTX). In normal conditions, treatment with BTX had no effects compared with the untreated condition (a, c). Co-treatment with TS and BTX inhibits therapeutic effect of TS on ALS phenotype of Mt TDP model (b, d–f). (F) Statistical analysis of **panel E**. Data was obtained from 8 control and 8 terbutaline sulfate and/or BTX-treated models.

We validated the potential therapeutic effect of TS in an *in vivo* zebrafish model of ALS, in which overexpression of mutant TDP-43 (Q331K) produces motor axon degeneration and defective neuromuscular junctions (NMJs)^15^. Treatment of the zebrafish containing TDP-43 mutant mRNA *Tg* (*olig2:dsred2*) with TS at 9 hours postfertilization (hpf), before the onset of axonal outgrowth, significantly prevented defects in axons and NMJ degeneration in the zebrafish model of ALS in a dose-dependent manner (*p* = 2.4 × e^−13^; Fig. 4A, B). Zebrafish injected with the mutant mRNA that were treated with 1 mM TS had virtually normal motor axons and NMJs. Moreover, TS was also able to recover function of dysregulated motor neurons in this model of ALS (Fig. 4C). Treatment of the zebrafish injected with TDP-43 with 1 mM TS at 36 hpf and 48 hpf, by which time axons and NMJ degeneration already occurred, significantly rescued motor axon and NMJ at 72 hpf (*p* = 2.1 × e^−11^; Fig. 4C, D).

Moreover, simultaneous treatment of the zebrafish model of ALS with butoxamine (BTX), a β_2_-adrenergic receptor antagonist, and TS (β_2_-adrenergic receptor agonist) resulted in motor axon defects similar to those of untreated zebrafish injected with mutant TDP-43 mRNA (*p* > 0.05; Fig. 4E, F). This suggests that cotreatment with BTX inhibits the therapeutic effect of TS on the TDP-43 mutation induced ALS-like phenotype of the zebrafish. Together, these data suggest that the therapeutic effect of TS on the TDP-43 mutation induced ALS-like phenotype in the zebrafish is mediated by activation of β_2_-adrenergic receptors.

## Discussion

Here we propose ClinDR as a method for predicting new indications for approved drugs based on known indications for similar drugs, and diseases inferred from both clinical signatures from large-scale EMR databases and genomics signatures. ClinDR outperformed previous approaches including models based on genomics similarity. We predicted 3,891 new indications for 226 drugs and 55 diseases, and the new indications significantly overlapped with the current clinical trials for new indications. Importantly, an *in vivo* validation of our predictions suggested that the asthma drug TS is a promising candidate for ALS treatment.

ALS is a lethal neurodegenerative disease with few therapeutic options. To our knowledge, riluzole is the only drug approved for ALS that presents prolonged survival trends, and there is a limited understanding of the related therapeutic mechanism^16^. Our study predicted an indication for the approved drug, TS, which is known as a β_2_-adrenergic receptor agonist, and has been used as a fast-acting bronchodilator. By co-treating our model of ALS with BTX, a β_2_-adrenergic receptor antagonist, we suggested that the efficacy of TS in our model might be associated with β_2_-adrenergic receptor activation.

ClinDR integrate diverse clinical and molecular-level signatures for drugs and diseases to generate drug-drug and disease-disease similarity. Zhang *et al*^17^ suggested an optimization method for integrating drug and disease associated signatures (called DDR) using different weightings for each of data sources, such as phenotypic terms and gene ontologies for interested drugs and diseases. Interestingly, Zhang and colleagues suggested phenotypic knowledge of drug and disease as major contributors to predict novel indications of drugs using their method. Zhang et al used knowledge-base information including known target proteins of drugs, phenotypic terms of disease and gene ontologies. In contract to this, ClinDR uses laboratory test results for drugs and diseases to detect phenotype associated signatures from human individuals and multiple molecular genetic signatures from public resources as well. The predictions using ClinDR are mainly based on similarities of drug-drug and disease-disease pairs via various clinical measures (i.e. physiological aspect) in human subjects. Currently, optimal integration of clinical measures with weighting values remain as challenging issues due to the heterogeneity of disease phenotype and drug responses in real clinical board. However, depends on our knowledge, ClinDR is an initial attempt for linking between human derived clinical (i.e, EMRs) and molecular-level signatures for drug repositioning.

We used laboratory test results to identify similarity of disease and drug pairs. Further analysis of EMRs may identify the relationships between the laboratory test results and patient phenotypes. The integration of multiple EMR databases across various hospitals remains a challenging issue. Nevertheless, our initial analysis of a single EMR database suggests that clinical records from EMRs are a promising resource for drug repositioning, and can be integrated with genomics data.

## Methods

### Dataset

The clinical data were derived from a 13-year inpatient EMR database at a tertiary teaching hospital, Ajou University Hospital in Korea. The EMR database included the admission date, discharge date, drug prescription, and laboratory test results from January 1, 1998 to March 31, 2010 (Table 1). The data were anonymized to protect patient privacy and confidentiality. The EMR analysis protocols were reviewed and approved by the Ajou University Hospital institutional review board. The hospital's information system allowed a patient's diagnosis and therapeutic records to be digitally recorded, and our database system had access to all hospital departments. The database contained >8,693 K drug prescriptions and >115 M laboratory test results from >1 M hospitalizations of 530 K individual patients. In a similar manner to previous work^4^, the genomic data were extracted from various databases including protein-protein interaction networks and gene ontology terms (see Supplemental methods for details).

### Similarity measures for drug- and disease-pair using clinical data

#### (i) Drug–drug similarity using EMR data

The EMR database contained drug prescription records, including the administration time points and various laboratory test results for patients during hospitalization. We tracked the administration records and any changes in the laboratory test results to profile the physiological variations in each test result after drug treatment by calculating the maximum differences, as described in our earlier studies^8^,^9^, as follows:
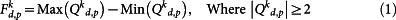
where *Q^k^_d,p_* represents the result for the *k-th* type of laboratory test for the *p-th* hospitalization case after *d-th* drug administration. Based on the maximum difference of *Q^k^_d,p_*, we computed the drug-induced change with the *d-th* drug treatment for the *k-th* type of laboratory test, *F^k^_d,p_*. Using a Wilcoxon rank sum test, we calculated the degree of similarity between the two drug-induced physiological distributions for a drug pair as the *p-*value for the corresponding laboratory test type. Finally, the normalized ranks of the *p-*values for all drug pairs were used as drug–drug similarity measures to reduce the heterogeneity of the *p-*value distributions for different laboratory tests. We assume that different laboratory tests may be related to specific physiological characteristics of distinct diseases or drugs. Thus, we calculated the similarity degree of disease or drug pairs using each test type separately. For the sparseness of laboratory test results, we here only used major types of laboratory test based on their high coverage of drug prescribed patient (≥0.3) having more than two test results during drug administration ordered (|*Q^k^_d,p_*| ≥ 2) (Table 2). Since only 1 K of cases prescribed one single drug, we selected cases which include less than five drug prescription records to maximize drug associated laboratory results with reduced expected disruptions of a drug induced laboratory test results by other drugs (Table 2).

#### (ii) Disease–disease similarity using EMR data

We compared the physiological state distributions in disease conditions with the laboratory test results before drug administration, as follows:
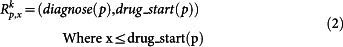
where *R^k^_p,x_* represents the result for the *k-th* type of laboratory test for the *p-th* hospitalization case at time *x*, and *diagnose*(*p*) indicates the disease condition of the *p-th* case. In addition, *drug_start*(*p*) represents the initial time of drug administration for case *p*. The time resolution of our EMR data was one day. Most drugs were prescribed after diagnosis, so we also included laboratory test results recorded on the same date as drug initiation; i.e., *x* ≤ *drug_start*(*p*). In equation (2), this study utilized diagnose(p) as a single diagnose code, which was assigned before the initial drug prescription recorded, and all of diagnose record missing cases were filtered in preprocess procedure of our EMR database. Although equation (2) determines various diagnose states including multi-morbidity condition, we independently utilized diagnose(p) as a single diagnose code for each case to generate distribution of disease associate laboratory test results to prepare larger number of cases for each disease. In a similar manner to the drug–drug similarity analysis, the normalized ranks of the Wilcoxon rank sum test *p-*values were used to generate a similarity matrix for all disease pairs.

### Similarity measures for drug- and disease-pair using genomic data

#### (i) Proportion of overlap between the PPI networks of drug-drug or disease-disease pairs

The PPI network modules of each drug or disease were explored using the drug or disease-related genes in our datasets (Supplemental methods). A drug or disease-related network was produced based on the first neighboring nodes of the seed genes. Based on our previous work, we determine similarity of disease related networks using normalized overlapping proportions of compared networks^18^. The statistical significance of our similarity measure was measured as the *p-*value based on the background distribution of 1000 randomly permuted tests. Finally, the normalized ranks of the *p-*values were used to represent the drug–drug or disease-disease similarity, with a range of [0, 1].

#### (ii) GO-based similarities of drug-drug or disease-disease pairs

The semantic similarity scores between drug or disease-related genes were quantified according to Resnik^19^. The similarity scores were transformed by rank normalization, with a range of [0, 1].

### Prediction of drug indications using similarity measures and the bipartite network of known drug–disease associations

ClinDR applied four steps to calculate the edge values using the similarity information based on: i) the clinical signatures, and ii) the genomic signatures; before iii) computing a final prediction value by integrating the edge scores from the genomic and clinical signatures; and iv) determining the edge label (i.e., true or false) using a given threshold. Suppose that we have a set of source drugs, *S* = {*s_1_*, *s_2_*, …, *s_m_*}, and a set of target diseases, *T* = {*t_1_*, *t_2_*, …, *t_n_*}. We add an edge *e_ij_* between drug *s_i_* and disease *t_j_* where a whole set of edges denotes a bipartite network of drugs and diseases *E* = {*e_11_*, …, *e_ij_*, …, *e_mn_*} with the corresponding binary labels of the edges *L*(*e_ij_*) (0 = false, 1 = true). ClinDR represents the edge label of a given drug–disease node pair using a classification rule *f*(*e_ij_*) > *θ* → *L*(*e_ij_*) = 1, where *f*(*e_ij_*) is the final predicted edge value. The detailed process used to compute *f*(*e_ij_*) was as follows.

#### (i) Calculating an edge score between a drug and a disease using the clinical data

Suppose that *SimLAB_S_*, and *SimLAB_T_* is the similarity matrix of all drug–drug and disease–disease pairs based on the clinical signatures (i.e. laboratory test results). There are various similarity measures based on different laboratory tests, so *SimLAB_S_* and *SimLAB_T_* are computed using the maximum similarity rank values among the different tests for individual drug or disease pairs. Thus, *SimLAB_S_*(*s_i_*, *s_p_*) means the similarity value between two drug nodes *s_i_* and *s_p_* (*s_i_*, *s_p_* ∈ *SimLAB_S_*) based on the clinical physiomic signatures, while *SimLAB_T_*(*t_i_*, *t_q_*) is the similarity value between two disease nodes *t_j_* and *t_q_* (*t_i_*, *t_q_* ∈ *SimLAB_T_*). Using similarities between disease-disease and drug-drug pairs, *P_c_* calculated edge scores between a queried pair of drug and disease (*s_i_* and *t_j_*) as follows:
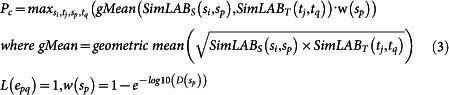
where *s_i_* ∈ *SimLAB_S_*, *t_j_* ∈ *SimLAB_T_*, *s_p_* ∈ *SimLAB_S_*, *t_q_* ∈ *SimLAB_T_*, and *L*(*e_pq_*) is an edge label between *s_p_* and *t_q_*. *L*(*e_pq_*) is 1 if there is a known drug indication between *s_p_* and *t_q_*, but otherwise it is 0. *D*(*s_p_*) is the degree of the drug node *s_p_* in a given bipartite network of drugs and diseases. Equation (3) calculates the maximum similarity for drugs and diseases in the known drug–disease association pairs by incorporating the degrees of the drug nodes. Since a drug having current clinical trial reports in *ClinicalTrial.gov* (http://clinicaltrials.gov/) displayed larger number of disease indications (*p-value* of Wilcoxon rank sum test = 2.27e-08), ClinDR gives weighting scores (*w*(*s_p_*)) for a drug node with various disease indication in equation (3) and (4), respectively. The equation of w(*s_p_*) was established by the distribution for the number of indications for known drugs, which have clinical trial reports as depicted in Supplemental Figure S5.

#### (ii) Calculating an edge score between a drug and a disease using genomic data

Suppose that *SimGEN_S_*, and *SimGEN_T_* is the similarity matrix of all drug–drug and disease–disease pairs based on the genomic signatures. Two types of genomic similarity measures can be derived from the GO terms and the PPI network analysis, *SimGEN_S_*, and *SimGEN_T_*, which are calculated using the maximum similarity rank value between them. Thus, *SimGEN_S_*(*s_i_*, *s_p_*) means the similarity value between two drug nodes *s_i_* and *s_p_* (*s_i_*, *s_p_* ∈ *SimGEN_S_*) based on the genomic signatures, while *SimGEN_T_*(*t_i_*, *t_q_*) is the similarity value between two disease nodes *t_j_* and *t_q_* (*t_i_*, *t_q_* ∈ *SimGEN_T_*). In a similar manner, we calculated the similarity-based *P_g_* (edge score between drug and disease) using genomic signatures of drugs and diseases:
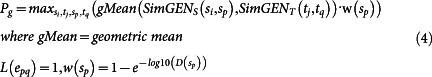
where *s_i_* ∈ *SimGEN_S_*, *t_j_* ∈ *SimGEN_T_*, *s_p_* ∈ *SimGEN_S_*, *t_q_* ∈ *SimGEN_T_*, and *L*(*e_pq_*) is an edge label between *s_p_* and *t_q_*. *L*(*e_pq_*) is 1 if there is a known drug indication between *s_p_* and *t_q_*, but otherwise it is 0.

#### (iii–iv) Final prediction of the edge value and label

Using the edge values predicted from the clinical and genomic signatures, ClinDR derived the final edge value by integrating the *P_c_* and *P_g_* scores. The final edge value *f*(*e_ij_*) was calculated using the following equation:
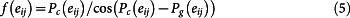

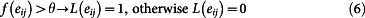
where *θ* is the threshold of the final edge value. The object of ClinDR is identification of similar drug and disease pairs among know drug-disease indications using clinical signatures (*P_c_*) and genomic features (*P_g_*) as well. In equation (5), the higher score is mainly derived by the larger *P_c_*, and minimum difference between *P_g_* and *P_c_* (*P_c_* – *P_g_*). We introduced cosine function to generate gradual determination of threshold for *f*(*e_ij_*) in equation (6) (*f*(*e_ij_*) > *θ*). The value range of f(eij) is from 0 to 1.8 based on our computational simulation. The value of *θ* was determined where ClinDR yielded maximum prediction performance in our 10-fold cross-validation scheme. *L*(*e_ij_*) has a Boolean value of 0 for false and 1 for true, depending on the drug indication between a drug and a disease. In the model comparison, genomic and clinical models had edge scores of either *P_g_* or *P_c_*.

### Prediction assessment and novel predictions

We used a 10-fold cross-validation to evaluate the performance of ClinDR using a prepared set of drugs and diseases (see Supplemental Methods for details).

### Experimental validation in zebrafish

Details of experimental validation used are in the Supplemental Methods.

## Supplementary Material

Supplementary Information supplementary information

## Figures and Tables

**Table 1:** Summary of the data used

Omics class	Features	Total no.
Clinical data	Total no. of cases	1,011,055^a^
	Total diagnosis code types (KCD6)	10,874
	Total diagnosis types (ICD10) with OMIM disease ids	425*
	Total no. of diagnosis records	2,788,135
	Total drug medication types (drug order/ATC codes)^b^	5,350/1,003
	No. of drug medication records	8,693,995
	Total laboratory test types	246
	Total no. of laboratory test records	9,494,169
	Total no. of cases with laboratory test records	313,347
	Mean laboratory tests per case	30.2
Genomic data	Total no. of diseases (OMIM disease ids)	17,986
	No. of disease-related genes	11,804
	No. of diseases that matched diagnosis codes (ICD10)	1,096
	Total drug types (ATC codes)^c^	1,615 (691**)
	No. of drug-related genes	14,466
	Total no. of protein–protein interactions^d^ (PPIs)	123,726
	Selected no. of PPIs (physical interactions^e^)	112,988
	Total no. of human genes	42,130
	No. of GO terms (related genes)	12,015 (17,919)
	No. of GO terms with evidence codes^f^ (related genes)	6,868 (8,671)

^a^One case was an admission–discharge event for a patient.

^b^The medication order was determined by the drug order code. The ATC code denotes the chemical compound name of a drug.

^c^Data resources: DrugBank (download date: 2011.10.23), CTD (download date: 2011.10.23), and STITCH (download date: 2011.10.23).

^d^Data resources: HPRD (download date: 2012.01.05), BioGrid (download date: 2011.12.27), IntAct (download date: 2011.11.20), MINT (download date: 2012.01.05), and DIP (download date: 2011.10.30).

^e^Physical interactions were determined by the PSI-MI codes: physical interaction (MI:0218), direct interaction (MI:0407), and physical association (MI:0915).

^f^GO evidence codes (EXP, IDA, IPI, IGI, and IEP).

*Total number of diagnosis codes used for further analysis of disease–disease similarities.

**Total number of drugs used for further analysis of drug–drug similarities.

**Table 2:** Summary of the similarity analysis of disease pairs and drug pairs

	Type of similarity	Features	Frequency
Clinical signatures	Disease–disease	Total laboratory test types^a^	246
		Selected laboratory test types^b^	11
		No. of laboratory tests at diagnosis points^c^	2,703,258 (408,722^d^)
	Drug–drug	Total laboratory test types	246
		Selected laboratory test types^e^	9
		No. of laboratory tests after medication^f^	9,494,169 (28,234^g^)
Genomic signatures	Disease–disease	Total no. of GO terms^h^	6,868
		Total no. of genes used in the network analysis^i^	11,804
	Drug–drug	Total no. of GO terms^h^	6,868
		Total no. of genes used in the network analysis^i^	14,466

^a^The laboratory test type was determined by the target protein or molecule detected by the serum/urine analysis, such as the total serum cholesterol concentration.

^b^We analyzed selected laboratory test results from 246 test types based on the total patient coverage (≥40%). Eleven laboratory tests were selected: erythrocyte sedimentation rate (EST), platelet count, activated partial thromboplastin time (aPTT), AC glucose value, and the GOT, GPT, alkaline phosphatase, total cholesterol, sodium, chloride, and total CO_2_ concentrations.

^c^The laboratory test results were prepared before the administration of drugs.

^d^Final number of results used. About 95% of the test results were filtered out because of the absence of matched OMIM disease IDs for diagnosis codes and a lack of patient coverage.

^e^The selection criteria were: 1) coverage of total drugs ≥30%; and 2) total observed cases >1,000. Nine laboratory test measures were selected: platelet count, AC glucose value, and the GOT, GPT, alkaline phosphatase, total cholesterol, sodium, chloride and total CO_2_ concentrations.

^f^The laboratory test results were prepared for drug-free and drug-treated patients.

^g^The laboratory results were selected based on these criteria: 1) assigned drug order codes with ATC (Anatomical Therapeutic Chemical classification system) codes and assigned PubChem IDs; 2) ≤5 drug treatments; and 3) the laboratory test points were prepared before and after drug administration events.

^h^In the present study, we determined the disease–disease and drug–drug similarities based on the distances between GO terms. The detailed methods used to calculate the GO-based similarity measures are described in the Methods section.

^i^In the present study, we determined the disease–disease and drug–drug similarities using a network-based membership scoring function. The detailed methods used to calculate this similarity measure are described in the Methods section.
