# Supplementary material for: Repurpose terbutaline sulfate for amyotrophic lateral sclerosis using electronic medical records
Source: Sci Rep. 2015 Mar 5;5:8580. doi: 10.1038/srep08580 (PMC4894399; doi:10.1038/srep08580)

Repurpose terbutaline sulfate for amyotrophic lateral sclerosis using electronic medical records

Hyojung Paik[1,4], Ah-Young Chung[2], Hae-Chul Park[2], Rae Woong Park[1], Kyoungho Suk[3], Jihyun Kim[1], Hyosil Kim[1], Atul J. Butte[4,*] and KiYoung Lee[1,]

[1]Department of Biomedical Informatics, Ajou University School of Medicine, Suwon, Gyeonggido, Korea
[2]Graduate School of Medicine, Korea University, Ansan, Gyeonggido, Korea
[3]Department of Pharmacology, Kyungpook National University, Daegu, Korea
[4]Department of Pediatrics, Stanford University School of Medicine, Stanford, CA, USA

[*] Correspondence should be addressed to abutte@stanford.edu (Fax:1-650-723-7070, Tel:1-650-723-3465)

Supplemental Methods

Dataset

Clinical physiomic data. We used a 13-year inpatient electronic medical record (EMR) database at a tertiary teaching hospital, Ajou University Hospital in Korea. The EMR database included the admission date, discharge date, drug prescription, and laboratory test results from January 1, 1998 to March 31, 2010 (**table S1**). The data were anonymized to protect patient privacy and confidentiality. The EMR analysis protocols were reviewed and approved by the Ajou University Hospital institutional review board. The hospital's information system allowed a patient's diagnosis and therapeutic records to be digitally

recorded, and our database system had access to all hospital departments. The database contained 8,693,995 drug prescriptions and 115,241,147 laboratory test results from 1,011,055 hospitalizations of 530,829 individual patients.

Genomic data. Genes related to drugs were extracted and compiled from DrugBank[1], Search Tool for Interactions of Chemicals (STITCH)[2], and Comparative Toxicogenomics Database (CTD)[3]. Disease-related genes were derived from the Online Mendelian Inheritance in Man database (OMIM, http://www.ncbi.nlm.nih.gov/omim). We integrated various well-known resources to prepare a human protein–protein interaction (PPI) network: Human Protein Reference Database, the Biological General Repository for Interaction Datasets, IntAct, the Molecular INTeraction database, and the Database of Interacting Proteins[4-8]. To produce valid PPI networks, we only used protein interactions with physical evidence; i.e., those with Proteomics Standard Initiative – Molecular Interactions (PSI-MI) codes, such as physical interactions (MI:0218), direct interactions (MI:0407), and physical associations (MI:0915). The Gene Ontology (GO) annotations of genes were downloaded from the NCBI website[9]. We only used GO annotations supported by experimental evidence codes: EXP (inferred from experiment), IDA (inferred from direct assay), IPI (inferred from physical interaction), IGI (inferred from genetic interaction), and IEP (inferred from expression pattern).

Generation of a bipartite network using known drug–disease associations. A bipartite network of diseases and drugs was constructed by assembling known drug–disease indications in Therapeutic Target Database (TTD)[10] and the paired drug administration and patient diagnosis records in our EMR database. To extract appropriate drug–disease indications, we excluded drug prescriptions for hospitalized cases with multiple diagnosis codes in our EMR database. Over 419,000 drug–disease associations were generated, and we

assigned metadata identifiers to each drug or disease, ATC codes to drugs, and ICD-10 and OMIM identifiers to diseases. After mapping the metadata identifiers of drugs and diseases, we established a standard set by filtering out drugs and diseases with null metadata identifiers. As the result 17,716 indications between 691 drugs and 425 diseases remained in the standard dataset for further analysis.

Disease diagnosis code classes. OMIM disease classes, such as metabolic diseases, were assigned based on a previous study of genomic level human disease classification[11].

Statistical and network analysis

In this study, R and MATLAB were used to perform statistical analyses including a Wilcoxon rank sum test, enrichment analysis, and a tenfold cross-validation analysis with various models. The network analysis, which included the graphical display of a drug–disease network, was performed using Cytoscape[12].

Prediction assessment

We used a tenfold cross-validation scheme to evaluate the performance of ClinDR using a prepared set of drugs and diseases. We selected 10% of the total drugs after removing the associated disease indications, and the test set was established by pairing all of the disease nodes with the selected drug nodes. The GBA method [13] was tested using the same set of drug–disease associations in the prepared drug–disease network. In most cases, we used the mean sensitivity, specificity, and AUC values as performance measures. For the GBA method, we calculated the mean sensitivity and specificity due to the deterministic nature of the GBA. The contributions of learning features, including the genomic and clinical

signatures, were analyzed using a feature elimination approach in the performance comparison.

The positive predictions of ClinDR included known and novel drug repositioning candidates. The predictions with known drug-disease associations were regarded as true positives, whereas others were treated as false positives. The possibilities of false positive predictions being candidates of novel repositioning were tested using drug–disease pairs from ongoing clinical trial reports owing to limited availability of true drug repositioning cases. The ongoing clinical trials were downloaded from a registry of federally and privately supported clinical trials, *ClinicalTrial.gov* (http://clinicaltrials.gov/), which were regarded as promising candidates for novel drug repositioning.

Experimental validation in zebrafish

Zebrafish lines. Tg(olig2:dsred2) zebrafish of either sex were used to visualize motor axons[14]. To block zebrafish pigmentation, 0.003% (w/v) 1- phenyl-2-thiourea (PTU) was added to the embryo medium (EM) at 24 hpf.

Chemical preparation and treatment. For the terbutaline sulfate treatment, embryos were incubated in EM containing 100 μM, 500 μM, and 1 mM terbutaline (hemi)sulfate (CAS No. 23031-32-5, Sigma-Aldrich), which were diluted from a 500 mM stock solution. For butoxamine hydrochloride treatment, embryos were incubated in EM containing 1 mM butoxamine hydrochloride (CAS No. 5696-15-1, Sigma-Aldrich) diluted from 200 mM stock solution.

Image analysis. Whole embryos were mounted on bridged slides containing 1.5% methylcellulose and imaged using a Zeiss Axio Imager M1 microscope equipped with a Zeiss

AxioCam MRc5 digital camera. For high-magnification *in vivo* imaging, embryos were anesthetized with 0.03% tricaine (Sigma-Aldrich) and mounted in 0.8% low-melting point agarose (SeaPlaque Agarose, Lonza) in glass-bottomed 35-mm dishes (MatTek). Fluorescent images were collected using an LSM510 laser scanning confocal microscope (Zeiss)

RNA synthesis and microinjections. Mutant human TDP-43 (TDP-43 Q331K) mRNA was produced using an mMESSAGE mMACHINE RNA Synthesis Kit (Ambion), followed by purification with a MEGAclear™ Kit (Ambion). Purified mutant human TDP-43 mRNA was diluted to a final concentration of 50 ng/μl, and 100–200 pg of mRNA was injected into one-cell stage *Tg*(*olig2:dsred2*) embryos.

REFERENCES

1. Knox, C. et al. DrugBank 3.0: a comprehensive resource for 'omics' research on drugs. *Nucleic acids research* **39**, D1035-1041 (2011).
2. Kuhn, M., von Mering, C., Campillos, M., Jensen, L.J. & Bork, P. STITCH: interaction networks of chemicals and proteins. *Nucleic acids research* **36**, D684-688 (2008).
3. Mattingly, C.J., Colby, G.T., Rosenstein, M.C., Forrest, J.N., Jr. & Boyer, J.L. Promoting comparative molecular studies in environmental health research: an overview of the comparative toxicogenomics database (CTD). *The pharmacogenomics journal* **4**, 5-8 (2004).
4. Peri, S. et al. Human protein reference database as a discovery resource for proteomics. *Nucleic acids research* **32**, D497-501 (2004).
5. Stark, C. et al. BioGRID: a general repository for interaction datasets. *Nucleic acids research* **34**, D535-539 (2006).
6. Kerrien, S. et al. The IntAct molecular interaction database in 2012. *Nucleic acids research* **40**, D841-846 (2012).
7. Zanzoni, A. et al. MINT: a Molecular INTeraction database. *FEBS letters* **513**, 135-140 (2002).
8. Xenarios, I. et al. DIP: the database of interacting proteins. *Nucleic acids research* **28**, 289-291 (2000).
9. Ashburner, M. et al. Gene ontology: tool for the unification of biology. The Gene Ontology Consortium. *Nature genetics* **25**, 25-29 (2000).
10. Chen, X., Ji, Z.L. & Chen, Y.Z. TTD: Therapeutic Target Database. *Nucleic acids research* **30**, 412-415 (2002).
11. Goh, K.I. et al. The human disease network. *Proc Natl Acad Sci U S A* **104**, 8685-8690 (2007).

12. Smoot, M.E., Ono, K., Ruscheinski, J., Wang, P.L. & Ideker, T. Cytoscape 2.8: new features for data integration and network visualization. *Bioinformatics* **27**, 431-432 (2011).
13. Chiang, A.P. & Butte, A.J. Systematic evaluation of drug-disease relationships to identify leads for novel drug uses. *Clinical pharmacology and therapeutics* **86**, 507-510 (2009).
14. Kucenas, S. et al. CNS-derived glia ensheath peripheral nerves and mediate motor root development. *Nature neuroscience* **11**, 143-151 (2008).

Repurpose terbutaline sulfate for amyotrophic lateral sclerosis using electronic medical records

Hyojung Paik[1,4], Ah-Young Chung[2], Hae-Chul Park[2], Rae Woong Park[1], Kyoungho Suk[3], Jihyun Kim[1], Hyosil Kim[1], Atul Butte[4*] and KiYoung Lee[1*]

[1]Department of Biomedical Informatics, Ajou University School of Medicine, Suwon, Gyeonggido, Korea
[2]Graduate School of Medicine, Korea University, Ansan, Gyeonggido, Korea
[3]Department of Pharmacology, Kyungpook National University, Daegu, Korea
[4]Department of Pediatrics, Stanford University School of Medicine, Stanford, CA, USA

[*] Correspondence should be addressed to abutte@stanford.edu
(Fax:1-650-723-7070, Tel:1-650-723-3465)

Supplemental Figures

Figure S1. Examples of clinical similarities using disease and drug related laboratory tests. (A) Similar diseases to acute nephritic syndrome (N00) using laboratory test results of erythrocyte sedimentation rate. Y-axis is the p-values of Wilcoxon rank sum tests and X-axis is the sorted diseases according to the p-values. **(B-D)** *P-values*+ were determined via hypergeometric test (N= total no, n= total no. of success, x= no of sampling bin, k = no of success in sampling bin). **(B-D)**Hierarchical clustering results for the erythrocyte sedimentation rate (N=20267, n=1056, x= 22, k=20) and total cholesterol (N=20267, n=1098, x=100, k=51) as examples of disease similarity. **(D)** Hierarchical clustering results for perturbation patterns of glutamic oxaloacetic transaminase as a example of drug similarity (N=1183, n=179, x=45, k=31).

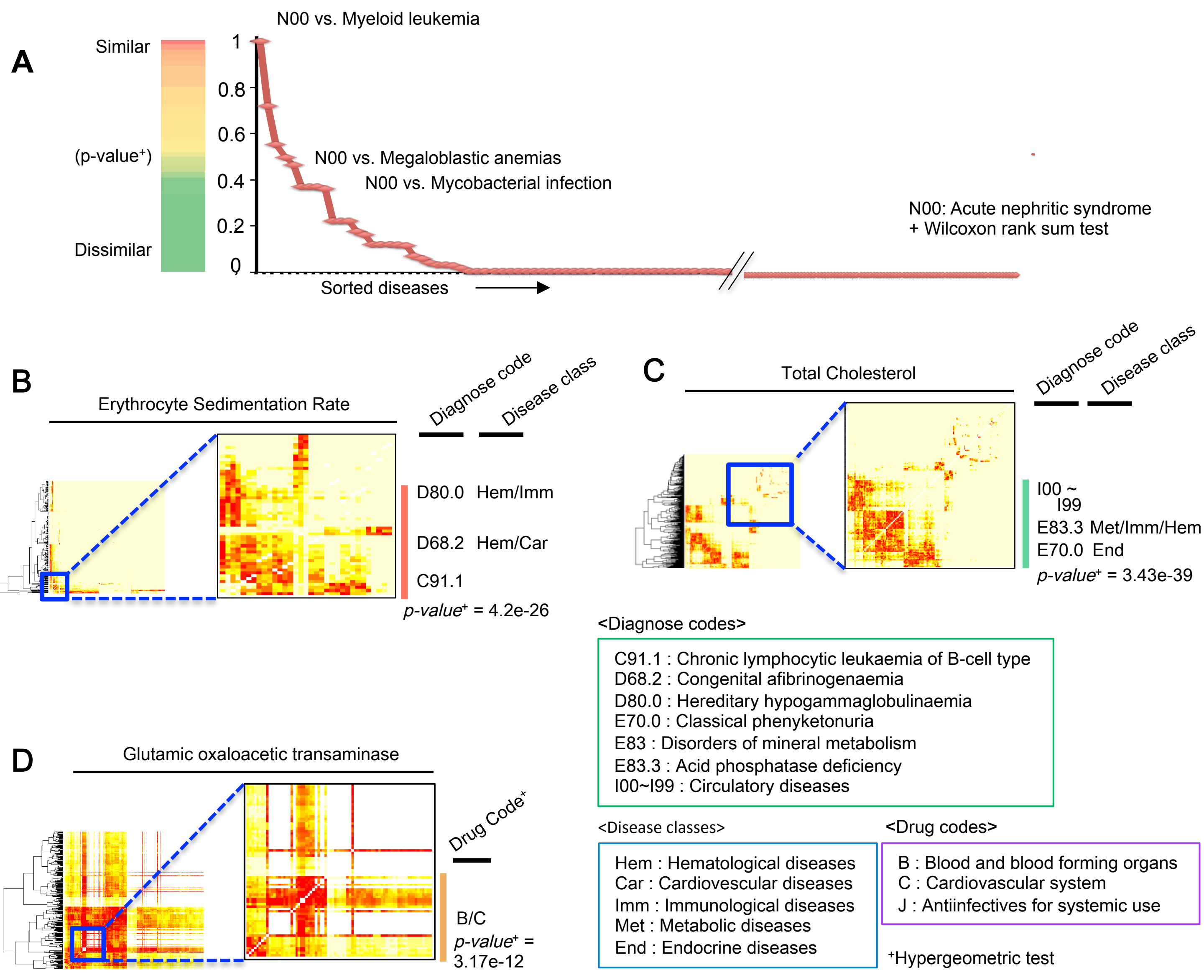

Figure S2. Network representation of predicted 3,891 novel indications between 226 drugs and 55 diseases. The left figure is the full drug-disease association network consisting positive predictions with ClinDR. The right figure is the subset of identified network of drug-disease associations including drugs which were currently reported for clinical trials for novel drug repositioning (blue line).

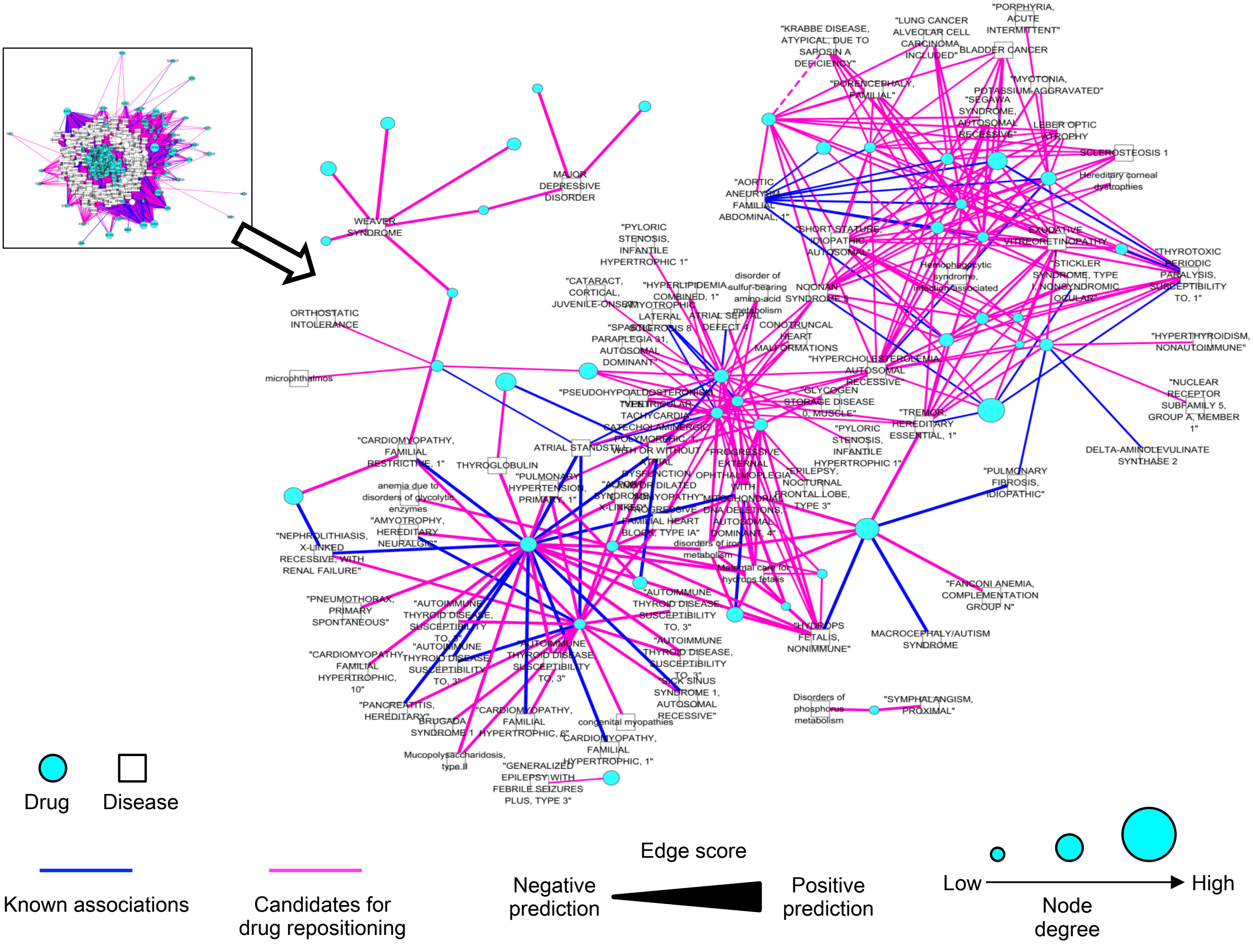

Figure S3. The overlap degree of ClinDR predictions with clinical trial reports. A hypergeometric test was performed using the set of novel indications in ongoing clinical trial reports and the false positive predictions of ClinDR.

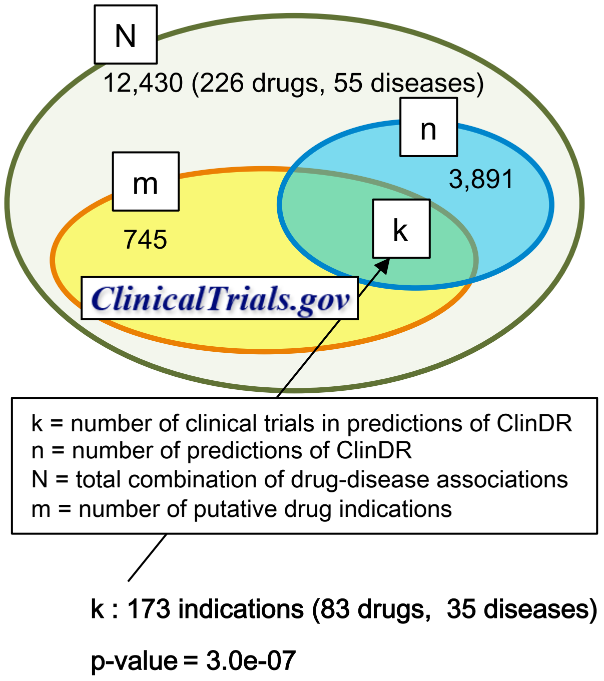

Figure S4. Drug class coverage for the predicted drug repositioning. ATC classes of drugs are represented as distinct colors.

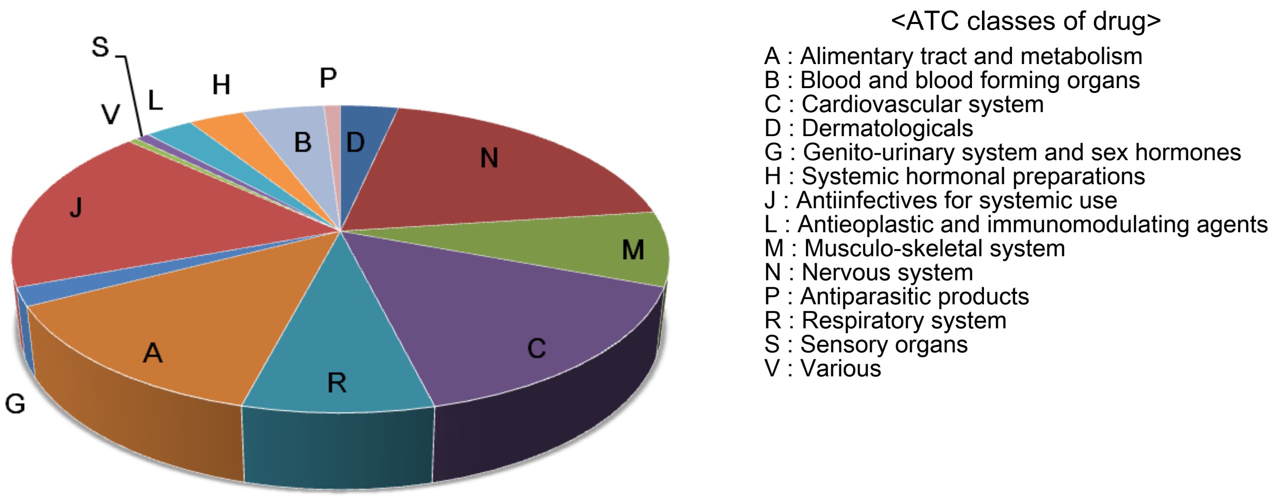

Figure S5. Weighting properties of known drug–disease associations for identifying drug repositioning candidates.

(A) Construction of a drug–disease association network using a large-scale EMR database and drug indications for known drug–disease associations in the Therapeutic Target Database. The set of drugs and diseases used for ClinDR training and evaluation are shown in the network on the left. **(B−E)** Drug node degree analysis for the known drug–disease network. **(B)** The known drug–disease indications and clinical trial cases are represented as a Venn diagram. We found that 226/1,114 approved drugs agreed with the clinical trial reports (i.e., $n(A \cap B) = 226$). **(C)** Comparison of the node degree distributions of drugs in ongoing clinical trials and other drugs (p = 2.27e–08; Wilcoxon signed rank test). **(D)** Bar chart showing the degrees of drug nodes in the two different approved drug groups. The pink bar represents putative repositioning targets in *ClinicalTrial.gov*, whereas the sky-blue bar represents the remaining drugs. **(E)** Normalized frequency ratio based on the total number of drugs in each degree interval. The red line denotes the approved drugs that have clinical trial reports, whereas the blue line represents the remaining approved drugs. **(F)** Overall trends of the degree weighting factors with ClinDR **(**green line**)**.

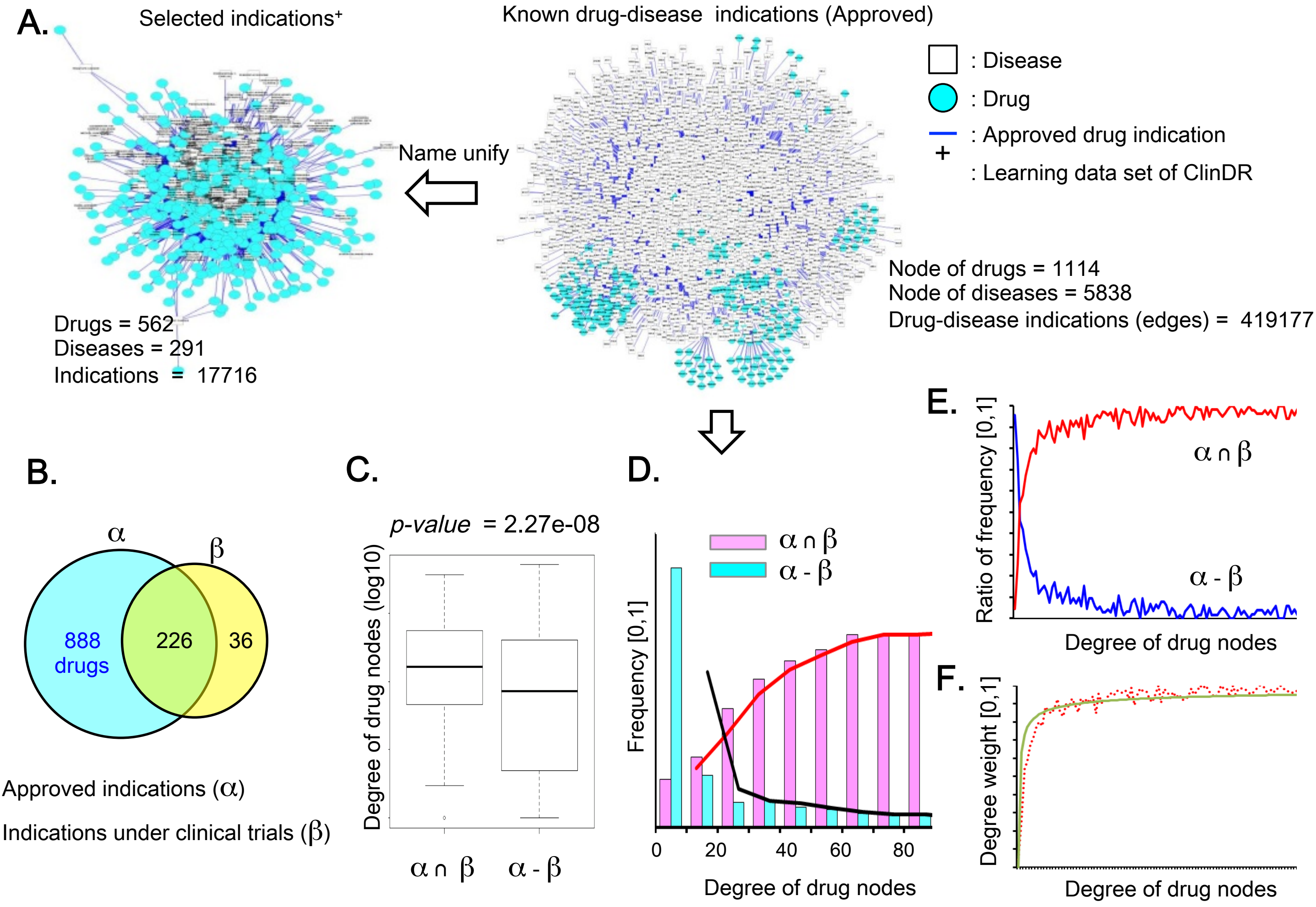

Supplement: Supplementary Information [file srep08580-s1.pdf]
